# Impact of Incorporating Pharmacy Technicians Into a Clinical Pharmacy Workflow on Aspirin Prescribing in Patients With Ischemic Vascular Disease

**DOI:** 10.1177/87551225261458228

**Published:** 2026-06-29

**Authors:** Licamied C. Macklin, Morgan P. Stewart, Kathryn P. Lin, Leticia R. Moczygemba, Leslie Simien

**Affiliations:** 1 CommUnityCare Health Centers, Austin, TX, USA; 2 CHRISTUS Spohn Hospital, Corpus Christi, TX, USA; 3 The University of Texas at Austin College of Pharmacy, USA

**Keywords:** aspirin, pharmacists, pharmacy technicians, cardiovascular diseases, outpatient

## Abstract

**Background:**

Federally qualified health centers (FQHCs) use core quality measures to guide care and secure funding. One core measure is “Ischemic Vascular Disease (IVD): Use of Aspirin or Another Antiplatelet.” A pilot program at an FQHC utilized the clinical pharmacy team to improve appropriate aspirin prescribing. Clinical pharmacy technicians identified eligible patients through a best practice advisory (BPA) in the electronic health record and contacted individuals with IVD not prescribed aspirin to schedule a clinical pharmacist appointment. Pharmacists then conducted medication reconciliations and ordered aspirin when indicated. This study evaluated the impact of incorporating pharmacy technicians into the clinical pharmacy workflow on the IVD quality measure.

**Methods:**

This retrospective chart review compared aspirin prescriptions ordered from June to November 2023 between patients contacted by pharmacy technicians and those who were not. Demographics, outreach attempts, aspirin orders, and diagnosis codes were collected. Descriptive statistics and Fisher’s exact test were used for analysis.

**Results:**

Pharmacy technicians attempted to contact 65 eligible patients, with 49 successfully reached. Aspirin was prescribed for 3.1% of control patients vs 30.6% in patients successfully contacted by the pharmacy team (*P* < .001). Resolved BPAs increased to 53.8% in the intervention group.

**Conclusion:**

A clinical pharmacy team significantly increased aspirin therapy among eligible patients with IVD, demonstrating the value of pharmacy team involvement on quality metrics. Future research should explore expanded integration of clinical pharmacy team services to enhance patient care.

## Background

Cardiovascular disease is the leading cause of death in the United States, accounting for 941 652 deaths in 2022.^1^ The use of antiplatelet agents is a cornerstone in the secondary prevention of atherosclerotic cardiovascular disease (ASCVD) and other ischemic vascular disease (IVD)-related conditions including individuals with a history of a myocardial infarction (MI), revascularization procedures, and stroke.^2^ Current US guidelines recommend low-dose aspirin 81 mg (75-100 mg) daily in patients with established ASCVD.^3^ Although use of aspirin has a well-defined role in secondary ASCVD prevention, real-world usage remains suboptimal.^4^

Federally qualified health centers (FQHCs) are community-based organizations that receive federal funding from the Bureau of Primary Health Care within the Health Resources and Services Administration (HRSA) to provide comprehensive medical services to underserved populations.^5^ To assess impact and quality of performance, all FQHCs provide an annual standardized quality report based on Uniform Data System (UDS) metrics.^6^ These measures enable benchmarking and can be used to compare facilities within each state and across the United States. One of the 2025 UDS clinical quality measures is entitled “Ischemic Vascular Disease (IVD): Use of Aspirin or Another Antiplatelet.” This measure captures the percentage of adults aged 18 years and older with a documented diagnosis of IVD, acute MI, coronary artery bypass graft, or percutaneous coronary intervention procedure who are prescribed aspirin or another antiplatelet.^7^ Although ASCVD is the preferred clinical term in contemporary guidelines, this report will use the term IVD to maintain consistency with UDS-defined metrics and FQHC reporting standards.

Clinical pharmacists and pharmacy technicians can play an essential role in ensuring patients with a history of IVD have access to and are taking optimal medication therapies.^8^ There is an upward trend in the use of pharmacy technicians in the clinical setting.^9-11^ The American Society of Health System Pharmacists’ (ASHP) Practice Advancement Initiative (PAI) 2030 recommends that pharmacy technicians “should participate in advanced roles in all practice settings to promote efficiency and improve access to patient care.”^12^ Many studies have found pharmacy technicians in clinical settings can improve pharmacist efficiency, but little has been published on a pharmacy technician-driven service to impact outpatient quality care metrics in an FQHC.^9^

The purpose of this study was to evaluate the impact of a clinical pharmacy team workflow on improving aspirin therapy rates among eligible patients with an IVD diagnosis at an FQHC.

## Methods

### Practice Setting

This study was conducted at a large FQHC in Central Texas. The clinical pharmacy department at the FQHC includes 2 clinical pharmacy technicians and 13 clinical pharmacists. Clinical pharmacists provide comprehensive medication management for chronic disease states, including IVD, via a collaborative practice agreement, which allows them to order medications, including aspirin, under a physician or advanced practice provider’s name. Clinical pharmacy technician roles were implemented at the FQHC in 2023 to support clinical pharmacy services and improve medication access. This includes aiding with prior authorizations, refills, administrative forms, and scheduling patients with clinical pharmacists to close quality metric care gaps such as smoking cessation or anticoagulation appointments. The clinic and the clinical pharmacy department see patients of all insurance status, including those who do not have health insurance and receive health care via the county’s Medical Access Program (MAP) available to those at or below 200% of the federal poverty level.^13^

### Clinic Workflow

**Figure 1.**
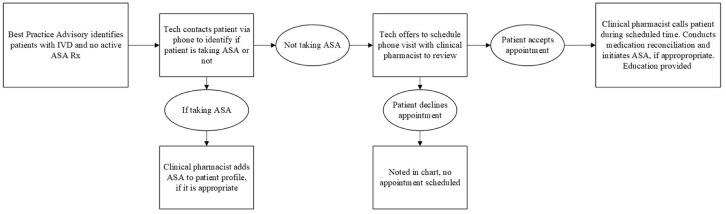
Clinical pharmacy team workflow. This figure illustrates the workflow of the pharmacy technician outreach and clinical pharmacist follow-up interventions. Pharmacy technicians attempted to contact each patient by telephone up to 2 times and left voicemail messages requesting a return call for each attempt. During outreach, pharmacy technicians used standardized call scripts and electronic medical record (EMR) documentation templates to assess current aspirin use and offer scheduling of a clinical pharmacist phone visit if appropriate. Abbreviations: ASA, aspirin; IVD, ischemic vascular disease; Rx, prescription order; Tech, clinical pharmacy technician.

A best practice advisory (BPA) to help meet the IVD: Use of Aspirin or Another Antiplatelet UDS metric was initiated at CommUnityCare Health Centers (CUC), an FQHC in Travis County, TX in May 2023. It identified patients with IVD-specific electronic health record (EHR)-mapped diagnosis codes who did not have an active prescription for aspirin (EHR-mapped codes listed in Supplemental Appendix 1). The clinical pharmacy department at CUC then developed a workflow to aid in the proper identification and assessment of eligible patients (Figure 1). All team members were trained on the protocol. The clinical pharmacy technicians ran a report of patients flagged via the BPA. They screened patient charts to ensure the BPA was active and attempted to contact patients via phone to identify if the patient was taking aspirin despite it not being documented in the chart. If the patient was not taking aspirin, they were offered an appointment with the clinical pharmacist to discuss aspirin appropriateness, counseling, and prescribing. If the patient was already taking aspirin, the technician sent a message to a clinical pharmacist who reviewed the chart to ensure it was indicated, added it to the patient’s chart, and alleviated the BPA. Technicians followed a basic script and documented their conversation in the chart using a standardized template developed by the clinical pharmacy team. The clinical pharmacy team also had a standardized template for clinical documentation.

### Study Design and Participants

This study was a retrospective chart review of data from June 1, 2023 through November 30, 2023. A master report was run through the EHR (Epic, Epic Systems Corporation), containing all patients who qualified for the BPA. The intervention group included patients whose charts were screened by the clinical pharmacy technician during the study period to confirm an active BPA. The control group was patients whose charts the clinical pharmacy technicians did not screen due to time. A random number generator was utilized to select the same number of control patients as the final intervention patient group and reviewed to ensure they met the inclusion criteria. Patients were included if they were on the master report, had a provider visit within the past year, and had a documented indication for aspirin in their problem list. Patients were excluded if they were deceased, did not have an indication listed on their problem list, or had an aspirin prescription on their profile unidentified by the report. Patient data were manually collected including demographic information, clinical pharmacy outreach attempts, aspirin ordering by clinical pharmacist and rationale, and diagnosis codes for indication of aspirin.

### Outcome Measures

The primary outcome was the number of prescriptions for aspirin ordered. Secondary outcomes included the number of patients flagged by the BPA but determined to be on aspirin therapy by clinical pharmacy technicians and the number of patients who accepted an appointment with a clinical pharmacist.

### Statistical Analysis

Descriptive statistics (mean, standard deviation, frequencies, and percentages) were used to describe all variables. Fisher’s exact test was used to compare the number of aspirin orders in the control group vs the intervention group. Comparisons were made with all patients the clinical pharmacy technician attempted to call and the subset of patients that the clinical pharmacy technician reached. This study was deemed exempt from the University of Texas at Austin Institutional Review Board.

## Results

**Figure 2.**
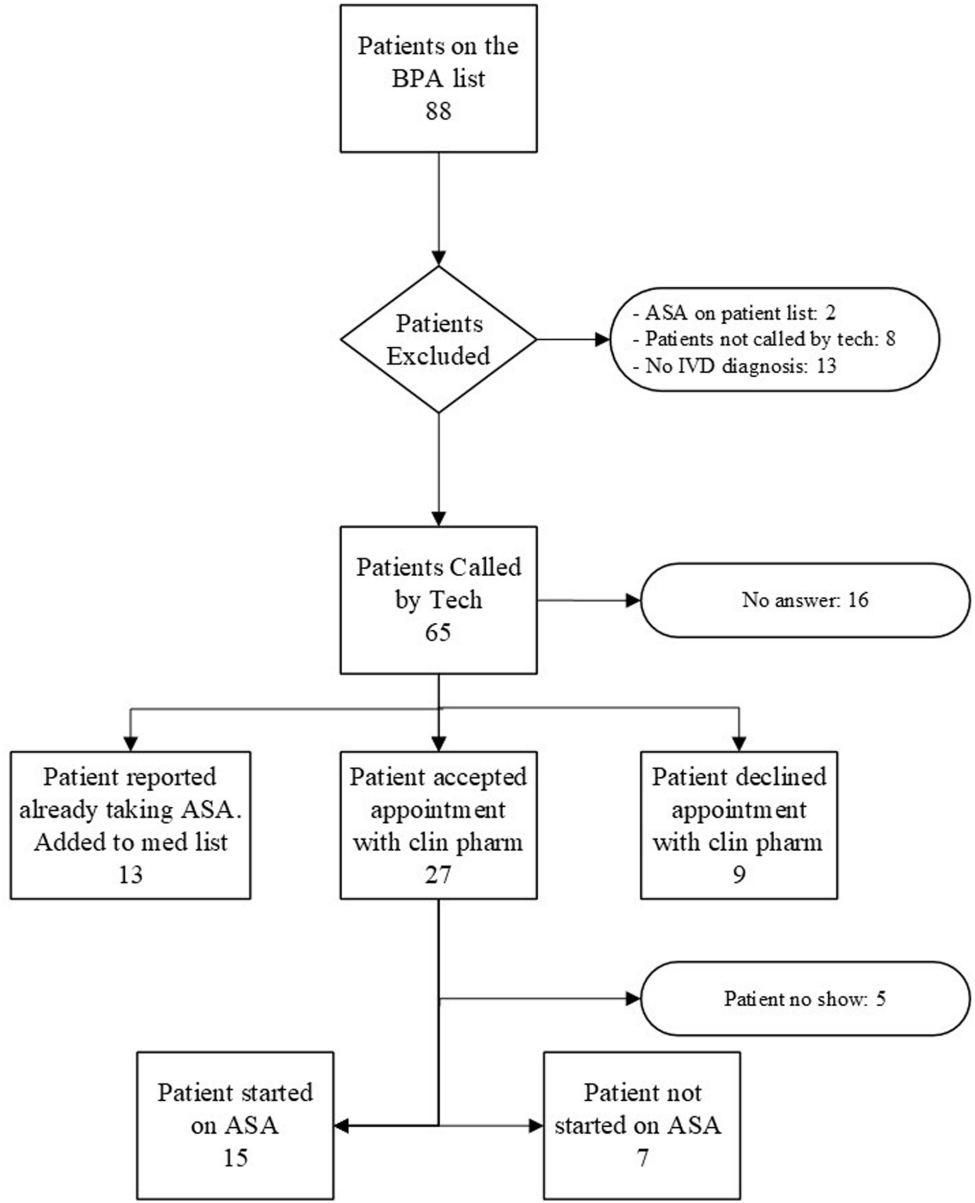
Patient flow diagram. This figure depicts the patient identification, exclusion, outreach, and outcomes associated with the pharmacy—led intervention. Numbers represent the total number of patients in each group. No show indicates the patient did not answer the phone for their appointment. Abbreviations: ASA, aspirin; BPA, best practice advisory; clin pharm, clinical pharmacist; med, medication; tech, clinical pharmacy technician.

The BPA identified 994 patients with a history of IVD but no active aspirin order. Within the study period, technicians had time to screen 88 patient charts (Figure 2). Fifteen patients were excluded from data analysis due to having an active aspirin order not caught by the BPA or a lack of qualifying IVD diagnosis, resulting in 73 eligible patients. Of these, pharmacy technicians attempted to call 65 (91.8%) patients. Others were not called due to time.

Baseline characteristics were generally similar between groups (Table 1). There were slightly more females in the control group compared with the intervention group (51% vs 49%). The majority of the patients in both groups self-identified as white. There were slightly more patients with MAP or private insurance in the control group compared with the intervention group. Most patients had a single IVD indication with more patients in the control group having atherosclerotic heart disease (61.5% vs 44.5%) and more cerebral infarction in the intervention group (29.2% vs 15.4%).

**Table 1.** Baseline Characteristics of All Participants.

Characteristic	Control group n,% (n = 65)	Intervention group (n = 65)
Sex		
Female	33 (51)	32 (49)
Male	32 (49)	33 (51)
Preferred language		
English	34 (52)	34 (52)
Spanish	26 (40)	26 (40)
Other*	5 (8)	5 (8)
Race		
White	47 (72)	49 (75)
Black	10 (15)	6 (9)
Other/unreported**	8 (12)	10 (15)
Ethnicity (%)		
Hispanic/Latino	30 (46)	35 (54)
Non-Hispanic/Latino	31 (47)	24 (37)
Unreported	4 (6)	6 (9)
Age (years, avg, std dev)	63.6 (11.8)	62 (11.0)
Payor source (%)		
Medicaid	9 (14)	9/65 (14)
Medicare	23 (35)	21/65 (32)
MAP	21 (32)	19/65 (29)
Private insurance	10 (15)	5/65 (8)
None	2 (3)	11/65 (17)
Number of IVD indications		
One	61 (94)	58 (89.2)
Two	4 (6)	7 (10.8)
IVD indication (ICD-10 code)		
Angina pectoris (I20.89, I20.9)	2 (3.1)	1 (1.5)
Non-ST elevation myocardial infarction (I21.4)	1 (1.5)	2 (3.1)
Atherosclerotic heart disease of native coronary artery without angina (I25.10)	40 (61.5)	29 (44.5)
History of myocardial infarction (I25.2, I25.29)	0 (0)	2 (3.1)
Atherosclerosis of coronary artery bypass graft (I25.810, Z95.1)	1 (1.5)	1 (1.5)
Cerebral infarction (I63.81, I63.9)	10 (15.4%)	19 (29.2)
Occlusion and stenosis of an artery (I65.02, I165.21, I65.29)	1 (1.5)	5 (7.7%)
Cerebral atherosclerosis, other cerebrovascular disease (I67.2, I67.850)	0 (0)	2 (3.1)
Sequelae of cerebral infarction, hemiplegia following cerebral infarction (I69.3, I69.351)	1 (1.5)	1 (1.5)
Transient ischemic attack (Z86.73, G45.9)	0 (0)	3 (4.6)
Atherosclerosis (I70.0, I70.1, I70.90, I70.203)	8 (12.3)	3 (4.6)
Peripheral vascular disease (I73.9)	3 (4.6)	1 (1.5)
Other^a^	0 (0)	4 (6.2)

* Other languages included 1 Vietnamese, 1 Arabic, 1 Nepali, 1 American sign language, and 1 Romanian.

** Other races included 5 unreported, 1 other Pacific Islander, and 2 Asian.

a Other IVD diagnoses with 1 patient each: coronary angioplasty status (history of PCI/stent) (Z98.61), other nonrheumatic aortic valve disorders (I35.8), celiac artery compression syndrome (vascular compression disorder) (I77.4), and the presence of prosthetic heart valve (Z95.2).

Among the 65 patients targeted for outreach, 16 patients (24.6%) did not answer the phone calls. Of the 49 patients successfully reached, 13 reported they were already taking aspirin, though it was not listed on their profile, 27 accepted an appointment with the clinical pharmacist, and 9 declined the appointment. One clinical pharmacist reviewed the charts of the 13 who reported home aspirin therapy, deemed it appropriate, and added it to the medication list to resolve the BPA. Of the 27 patients who accepted an appointment with a clinical pharmacist, 22 attended. Among those who attended, 15 patients (68.2%) were initiated on aspirin, while 7 (31.8%) were not started due to lack of true indication (n = 6) and patient deferral of the decision (n = 1).

**Figure 3.**
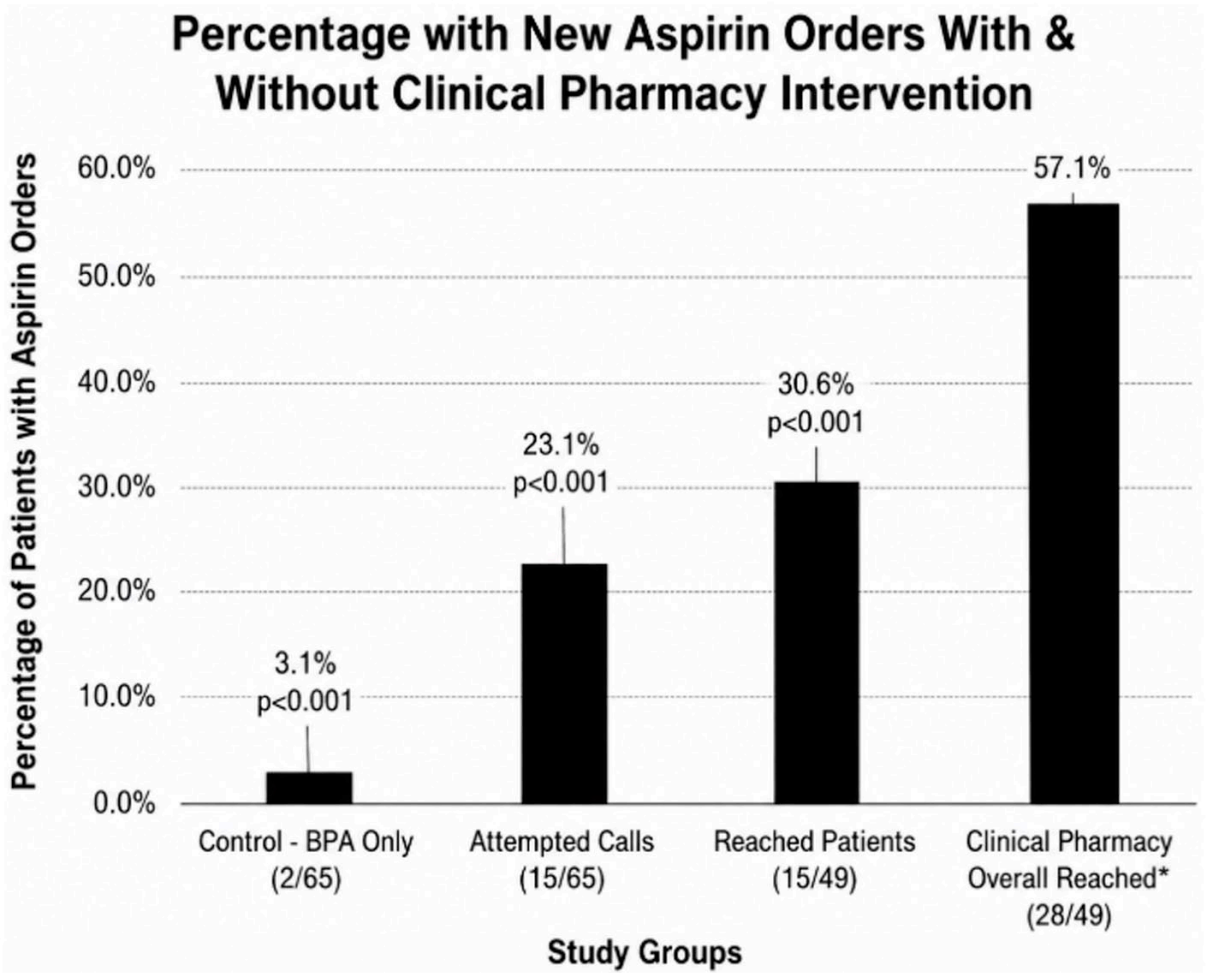
Percentage of patients with aspirin orders with and without clinical pharmacy intervention. The percentage of patients with aspirin orders placed by a clinical pharmacist increased significantly following implementation of the intervention compared with the control group (*P* < .001). The highest prescribing rate was observed among patients successfully reached by the pharmacy technician. *Overall reached includes patients who were already taking aspirin but it was added to the chart by the pharmacist and patients who were newly initiated on aspirin by a clinical pharmacist.

Overall, clinical pharmacists placed 28 new aspirin orders and resolved BPAs increased from 0% to 53.8% in the intervention group. Among the 65 patients in the control group, only 2 (3.1%) were prescribed aspirin during the study period. The percentage of patients newly prescribed aspirin increased from 0% to 3.1% (2/65) in the control group with a BPA only compared with 23.1% (15/65) in the intervention group (*P* < .001 for each). In the subset of patients, the clinical pharmacy technician reached by phone, this average was even higher, 30.6% (15/49, *P* < .001) (Figure 3).

## Discussion

Use of pharmacy technicians in the clinical pharmacy workflow increased true aspirin prescribing from 3.1% using the BPA alone to 30.6% after implementation of the BPA and clinical pharmacy technician workflow, which demonstrated a statistically significant difference. An even larger impact was demonstrated in the improved quality metric for the number of individuals with a history of IVD who were already taking aspirin but did not have this documented in the EHR (57.1%). The impact of the clinical pharmacy team’s intervention is noteworthy at a systems level, given the funding ramifications for FQHC systems and reporting quality indicators. Uniform Data System measure performance is incorporated into scoring criteria for HRSA quality recognition badges and budget renewal applications. Sustained improvement, or underperformance, can directly affect a health center’s competitiveness for grant funding.^6^ Demonstrating a replicable, team-based intervention capable of moving these metrics is relevant to health centers seeking to strengthen patient outcomes and institutional standing. The results of this study are consistent with previous research, highlighting the importance of utilizing pharmacy technicians to their full potential.^9-11^ The novel aspect of this study is the utilization of a clinical pharmacy team to impact UDS metrics, which are used to assess care quality and guide future quality-improvement efforts.^6^

Importantly, while aspirin prescribing rates were highest among patients successfully reached by the pharmacy technician, evaluation of the full cohort of patients targeted for outreach (n = 65) provides a more comprehensive assessment of the intervention’s real-world feasibility and sustainability. Pharmacy technician time was required to screen and attempt outreach for all patients, regardless of successful contact, and thus, the overall yield reflects the true operational investment of this workflow. This cohort found a high rate of success of contacting patients. At the same time, the higher prescribing rate among reached patients underscores the effectiveness of direct patient engagement and suggests that improving contact rates may further enhance the impact of this intervention.

This study also highlights important limitations of relying on BPA-driven identification alone. In the BPA-only cohort, despite BPA identification of eligible patients, aspirin prescribing occurred in only a small proportion of patients, demonstrating that passive clinical decision support may be insufficient to close care gaps. Best practice advisory-based approaches depend on patients presenting for care and providers having adequate time and prioritization to act on alerts during visits. In busy primary care settings, more acute concerns may take precedence, and preventative interventions such as aspirin initiation may be deferred due to competing demands or clinical inertia. Although we did not review the control group to determine whether they had a visit during the study period, the large number of patients identified in this group without appropriate therapy further supports this gap. In contrast, the clinical pharmacy workflow represents a proactive, team-based approach that ensures identification, outreach, and follow-up independent of scheduled visits, thereby reducing missed opportunities for intervention and improving timely delivery of guideline-directed care.

This study has a few notable strengths, including its real-world implementation using objective patient identification through ICD-10 codes. The use of objective patient identification enhances the study’s reproducibility and supports its applicability across diverse clinical settings. A comparison of preintervention and postintervention data allows for assessment of change over time.

There are also several limitations to the study. First, the study had a small study population in relation to the number of eligible patients who could be contacted during the short time frame. This is likely due to not having dedicated clinic time for the clinical pharmacy technicians to contact patients as technicians were expected to continue their day-to-day duties in addition to this initiative. Therefore, this pilot program’s reach was limited relative to the total eligible population. This limits the external validity of this program. Identifying and addressing structural constraints is essential before broader program deployment and applicability of this model to other institutions. There was also inconsistent technician documentation with occasional findings of pharmacists notating technicians who had contacted a patient, but no documentation from the technician, so they may not have been included in this study. Again, this could be due to the limited dedicated time for this project and the need for further clarification of the workflow with the team. Several implementation lessons have been identified that may guide future efforts to the meaningful expansion of the program’s reach. Scheduled time blocks dedicated for technician outreach may increase the number of eligible patients contacted. Prioritizing outreach during lower-volume clinic periods, developing a standardized call script to improve efficiency and consistency, and establishing explicit documentation expectations for all pharmacy team members are strategies currently under consideration.

One of the main concepts of this study involves acknowledgment of the promising new role of clinical pharmacy technicians within the workflow alongside clinical pharmacists. Utilizing pharmacy technicians effectively can increase efficiency and productivity within the workplace, and this study is a great example of this. Navigating the patients on this report and contacting them regarding their current aspirin therapy allow clinical pharmacists to continue providing exceptional primary care to their daily scheduled patients without administrative burden. By clinical pharmacy technicians identifying the patients who were already on aspirin, they allowed more time for the clinical pharmacists to provide thorough assessments on others who needed it. The additional contact and engagement with the team may also have had a positive impact on the patient’s willingness to take the aspirin, though patient data would be needed to confirm this.

## Conclusion

The results demonstrate that the implementation of a clinical pharmacy team improved the amount of eligible patients with IVD prescribed aspirin from 2% to 30% in the span of 5 months. This study identified a positive impact of a clinical pharmacy team on quality metrics. Future studies can focus on workflow improvement and the expansion of pharmacy technician roles to enhance clinical pharmacy-driven outcomes.

## Supplemental Material

sj-docx-1-pmt-10.1177_87551225261458228 – Supplemental material for Impact of Incorporating Pharmacy Technicians Into a Clinical Pharmacy Workflow on Aspirin Prescribing in Patients With Ischemic Vascular Disease Supplemental material, sj-docx-1-pmt-10.1177_87551225261458228 for Impact of Incorporating Pharmacy Technicians Into a Clinical Pharmacy Workflow on Aspirin Prescribing in Patients With Ischemic Vascular Disease by Licamied C. Macklin, Morgan P. Stewart, Kathryn P. Lin, Leticia R. Moczygemba and Leslie Simien in Journal of Pharmacy Technology
