# Supplementary material for: Impact of Incorporating Pharmacy Technicians Into a Clinical Pharmacy Workflow on Aspirin Prescribing in Patients With Ischemic Vascular Disease
Source: J Pharm Technol. 2026 Jun 29:87551225261458228. Online ahead of print. doi: 10.1177/87551225261458228 (PMC13314662; doi:10.1177/87551225261458228)
Supplement: sj-docx-1-pmt-10.1177_87551225261458228 – Supplemental material for Impact of Incorporating Pharmacy Technicians Into a Clinical Pharmacy Workflow on Aspirin Prescribing in Patients With Ischemic Vascular Disease [file sj-docx-1-pmt-10.1177_87551225261458228.docx]

**Supplemental:**

Complete List of Electronic Medical Record-Mapped Codes with Associated Ischemic Vascular Disease Diagnoses

| Mapped Codes | Associated Diagnosis |
| --- | --- |
| 1055001 | Stenosis of precerebral artery (disorder) |
| 4557003 | Preinfarction syndrome (disorder) |
| 5534004 | Senile arteritis (disorder) |
| 14977000 | Multiple AND bilateral precerebral artery thrombosis (disorder) |
| 19057007 | Status anginosus (disorder) |
| 20059004 | Occlusion of cerebral artery (disorder) |
| 21470009 | Syncope anginosa (disorder) |
| 23687008 | Coronary artery spasm (disorder) |
| 25106000 | Impending infarction (disorder) |
| 26900001 | Coronary ostium stenosis (disorder) |
| 28790007 | Obstruction of precerebral artery (disorder) |
| 29899005 | Coronary artery embolism (disorder) |
| 35928006 | Nocturnal angina (disorder) |
| 37943007 | Multiple AND bilateral precerebral artery embolism (disorder) |
| 39823006 | Generalized atherosclerosis (disorder) |
| 40276003 | Embolism of precerebral artery (disorder) |
| 41334000 | Angina, class II (disorder) |
| 48601002 | Thrombosis of precerebral artery (disorder) |
| 49176002 | Arteriosclerotic gangrene (disorder) |
| 50808002 | Atheroma of artery (disorder) |
| 51274000 | Atherosclerosis of arteries of the extremities (disorder) |
| 51677000 | Atheroembolism of renal arteries (disorder) |
| 52674009 | Ischemia (disorder) |
| 53741008 | Coronary arteriosclerosis (disorder) |
| 54519002 | Basilar artery stenosis (disorder) |
| 54687002 | Arterial embolism (disorder) |
| 55382008 | Cerebral atherosclerosis (disorder) |
| 57357009 | Transient ischemia (disorder) |
| 59021001 | Angina decubitus (disorder) |
| 59062007 | Coronary stricture (disorder) |
| 60989005 | Thromboembolism of renal arteries (disorder) |
| 61490001 | Angina, class I (disorder) |
| 63739005 | Coronary occlusion (disorder) |
| 64586002 | Carotid artery stenosis (disorder) |
| 64775002 | Vertebral artery thrombosis (disorder) |
| 65084004 | Vertebral artery embolism (disorder) |
| 65198009 | Arterial thrombosis (disorder) |
| 66189004 | Postmyocardial infarction syndrome (disorder) |
| 67682002 | Coronary artery atheroma (disorder) |
| 67992007 | Multiple AND bilateral precerebral artery obstruction (disorder) |
| 69742007 | Monckeberg's medial sclerosis (disorder) |
| 71444005 | Cerebral arterial thrombosis (disorder) |
| 72092001 | Arteriosclerotic vascular disease (disorder) |
| 73192008 | Multiple AND bilateral precerebral artery stenosis (disorder) |
| 75543006 | Cerebral embolism (disorder) |
| 80383008 | Embolism of iliac artery (disorder) |
| 80606009 | Carotid artery embolism (disorder) |
| 81817003 | Atherosclerosis of aorta (disorder) |
| 85284003 | Angina, class III (disorder) |
| 87343002 | Prinzmetal angina (disorder) |
| 88174006 | Basilar artery thrombosis (disorder) |
| 89323001 | Angina, class IV (disorder) |
| 90520006 | Vertebral artery stenosis (disorder) |
| 92517006 | Calcific coronary arteriosclerosis (disorder) |
| 95458005 | Cerebellar artery occlusion (disorder) |
| 95459002 | Cerebellar artery thrombosis (disorder) |
| 95580006 | Renal artery embolism (disorder) |
| 109381003 | Sclerosis of saphenous vein (disorder) |
| 111291001 | Senile endarteritis (disorder) |
| 111296006 | Basilar artery embolism (disorder) |
| 111298007 | Chronic cerebral ischemia (disorder) |
| 111299004 | Atheroma of cerebral arteries (disorder) |
| 123641001 | Left coronary artery occlusion (disorder) |
| 123642008 | Right coronary artery occlusion (disorder) |
| 129573006 | Atherosclerotic occlusive disease (disorder) |
| 194823009 | Acute coronary insufficiency (disorder) |
| 194828000 | Angina (disorder) |
| 194842008 | Single coronary vessel disease (disorder) |
| 194843003 | Double coronary vessel disease (disorder) |
| 194849004 | Generalized ischemic myocardial dysfunction (disorder) |
| 195182007 | Vertebral artery occlusion (disorder) |
| 195183002 | Multiple and bilateral precerebral arterial occlusion (disorder) |
| 195185009 | Cerebral infarct due to thrombosis of precerebral arteries (disorder) |
| 195186005 | Cerebral infarction due to embolism of precerebral arteries (disorder) |
| 195189003 | Cerebral infarction due to thrombosis of cerebral arteries (disorder) |
| 195190007 | Cerebral infarction due to embolism of cerebral arteries (disorder) |
| 195200006 | Carotid artery syndrome hemispheric (disorder) |
| 195206000 | Intermittent cerebral ischemia (disorder) |
| 195230003 | Cerebral infarction due to cerebral venous thrombosis, non-pyogenic (disorder) |
| 195231004 | Occlusion and stenosis of cerebral arteries, not resulting in cerebral infarction (disorder) |
| 195233001 | Occlusion and stenosis of anterior cerebral artery (disorder) |
| 195234007 | Occlusion and stenosis of posterior cerebral artery (disorder) |
| 195236009 | Occlusion and stenosis of multiple and bilateral cerebral arteries (disorder) |
| 195254008 | Extremity artery atheroma (disorder) |
| 195317001 | Embolism and thrombosis of the thoracic aorta (disorder) |
| 195319003 | Embolism and thrombosis of the brachial artery (disorder) |
| 195320009 | Embolism and thrombosis of the radial artery (disorder) |
| 195321008 | Embolism and thrombosis of the ulnar artery (disorder) |
| 195323006 | Embolism and thrombosis of the femoral artery (disorder) |
| 195324000 | Embolism and thrombosis of the popliteal artery (disorder) |
| 195325004 | Embolism and thrombosis of the anterior tibial artery (disorder) |
| 195326003 | Embolism and thrombosis of the dorsalis pedis artery (disorder) |
| 195327007 | Embolism and thrombosis of the posterior tibial artery (disorder) |
| 195340002 | Embolism and thrombosis of the splenic artery (disorder) |
| 195341003 | Embolism and thrombosis of the axillary artery (disorder) |
| 195342005 | Embolism and thrombosis of the celiac artery (disorder) |
| 195343000 | Embolism and thrombosis of hepatic artery (disorder) |
| 225566008 | Ischemic chest pain (finding) |
| 230692004 | Infarction - precerebral (disorder) |
| 230693009 | Anterior cerebral circulation infarction (disorder) |
| 230694003 | Total anterior cerebral circulation infarction (disorder) |
| 230695002 | Partial anterior cerebral circulation infarction (disorder) |
| 230698000 | Lacunar infarction (disorder) |
| 230699008 | Pure motor lacunar infarction (disorder) |
| 230700009 | Pure sensory lacunar infarction (disorder) |
| 230701008 | Pure sensorimotor lacunar infarction (disorder) |
| 230702001 | Lacunar ataxic hemiparesis (disorder) |
| 230703006 | Dysarthria-clumsy hand syndrome (disorder) |
| 230716006 | Carotid territory transient ischemic attack (disorder) |
| 233817007 | Triple vessel disease of the heart (disorder) |
| 233819005 | Stable angina (disorder) |
| 233821000 | New onset angina (disorder) |
| 233823002 | Silent myocardial ischemia (disorder) |
| 233844002 | Accelerated coronary artery disease in transplanted heart (disorder) |
| 233955003 | Abdominal aortic atherosclerosis (disorder) |
| 233956002 | Aortoiliac atherosclerosis (disorder) |
| 233958001 | Peripheral ischemia (disorder) |
| 233959009 | Upper limb ischemia (disorder) |
| 233960004 | Critical upper limb ischemia (disorder) |
| 233961000 | Lower limb ischemia (disorder) |
| 233962007 | Critical lower limb ischemia (disorder) |
| 233964008 | Internal carotid artery stenosis (disorder) |
| 233970002 | Coronary artery stenosis (disorder) |
| 233972005 | Aortic bifurcation embolus (disorder) |
| 233973000 | Femoral artery embolus (disorder) |
| 233974006 | Brachial artery embolus (disorder) |
| 236120009 | Ischemia of stoma (disorder) |
| 251024009 | Coronary graft stenosis (disorder) |
| 266253001 | Precerebral arterial occlusion (disorder) |
| 266254007 | Occlusion of carotid artery (disorder) |
| 266262004 | Arterial embolus and thrombosis (disorder) |
| 266263009 | Embolism and thrombosis of the abdominal aorta (disorder) |
| 274101000 | Aortic thromboembolism (disorder) |
| 276219001 | Occipital cerebral infarction (disorder) |
| 280871000 | Vascular degeneration (disorder) |
| 281091000 | Ischemic myocardial dysfunction (disorder) |
| 286959000 | Peripheral arterial embolism (disorder) |
| 287731003 | Cerebral ischemia (disorder) |
| 297136002 | Axillary artery embolus (disorder) |
| 297138001 | Embolus of circle of Willis (disorder) |
| 297141005 | Popliteal artery embolus (disorder) |
| 300917007 | Ischemia of feet (disorder) |
| 300920004 | Carotid atherosclerosis (disorder) |
| 300995000 | Exercise-induced angina (disorder) |
| 301755001 | Ischemic foot (disorder) |
| 302728008 | Endarteritis deformans (disorder) |
| 302904002 | Infarction of visual cortex (disorder) |
| 302910002 | Atherosclerotic renal artery stenosis (disorder) |
| 302930003 | Endarteritis obliterans (disorder) |
| 307363008 | Multiple lacunar infarcts (disorder) |
| 307406004 | Trash foot (disorder) |
| 307407008 | Ischemic hand (disorder) |
| 307408003 | Ischemic toe (disorder) |
| 307409006 | Ischemic finger (disorder) |
| 307766002 | Left sided cerebral infarction (disorder) |
| 307767006 | Right sided cerebral infarction (disorder) |
| 312375001 | Upper limb arterial embolus (disorder) |
| 312378004 | Lower limb arterial embolus (disorder) |
| 312822006 | Critical ischemia of foot (disorder) |
| 314116003 | Post infarct angina (disorder) |
| 315025001 | Refractory angina (disorder) |
| 315348000 | Asymptomatic coronary heart disease (disorder) |
| 361132001 | Senile arteriosclerosis (disorder) |
| 361133006 | Arteriosclerosis obliterans (disorder) |
| 363340006 | Vascular disorder of extremity (disorder) |
| 371039008 | Thromboembolic disorder (disorder) |
| 371041009 | Embolic stroke (disorder) |
| 371803003 | Multi vessel coronary artery disease (disorder) |
| 371804009 | Left main coronary artery disease (disorder) |
| 371805005 | Significant coronary bypass graft disease (disorder) |
| 371806006 | Progressive Angina (disorder) |
| 371807002 | Atypical angina (disorder) |
| 371808007 | Recurrent angina status post percutaneous transluminal coronary angioplasty (disorder) |
| 371809004 | Recurrent angina status post coronary stent placement (disorder) |
| 371810009 | Recurrent angina status post coronary artery bypass graft (disorder) |
| 371811008 | Recurrent angina status post rotational atherectomy (disorder) |
| 371812001 | Recurrent angina status post directional coronary atherectomy (disorder) |
| 394659003 | Acute coronary syndrome (disorder) |
| 402861007 | Ischemic gangrene (disorder) |
| 408546009 | Coronary artery bypass graft occlusion (disorder) |
| 408665008 | Pontine artery thrombosis (disorder) |
| 413102000 | Infarction of basal ganglia (disorder) |
| 413439005 | Acute ischemic heart disease (disorder) |
| 413444003 | Acute myocardial ischemia (disorder) |
| 413552002 | Anterior segment ischemia (disorder) |
| 413758000 | Cardioembolic stroke (disorder) |
| 413838009 | Chronic ischemic heart disease (disorder) |
| 413844008 | Chronic myocardial ischemia (disorder) |
| 414545008 | Ischemic heart disease (disorder) |
| 414795007 | Myocardial ischemia (disorder) |
| 420006002 | Obliterative coronary artery disease (disorder) |
| 425527003 | Atheromatous embolus of lower limb (disorder) |
| 426107000 | Acute lacunar infarction (disorder) |
| 426651005 | Occlusion of bilateral carotid arteries (disorder) |
| 427296003 | Thalamic infarction (disorder) |
| 427567003 | Atheromatous embolus of upper limb (disorder) |
| 428196007 | Mixed myocardial ischemia and infarction (disorder) |
| 428507003 | Atherosclerotic stenosis of brachiocephalic artery (disorder) |
| 429245005 | Recurrent coronary arteriosclerosis after percutaneous transluminal coronary angioplasty (disorder) |
| 429559004 | Typical angina (disorder) |
| 429673002 | Arteriosclerosis of coronary artery bypass graft (disorder) |
| 432083006 | Occlusive disease of artery of lower extremity (disorder) |
| 432504007 | Cerebral infarction (disorder) |
| 441574008 | Atherosclerosis of artery (disorder) |
| 442224005 | Arteriosclerosis of autologous vein coronary artery bypass graft (disorder) |
| 442240008 | Arteriosclerosis of nonautologous coronary artery bypass graft (disorder) |
| 442421004 | Arteriosclerosis of arterial coronary artery bypass graft (disorder) |
| 442439008 | Atherosclerosis of bypass graft of limb (disorder) |
| 442701004 | Atherosclerosis of nonautologous biological bypass graft of limb (disorder) |
| 443502000 | Atherosclerosis of coronary artery (disorder) |
| 443971004 | Arteriosclerosis of artery of extremity (disorder) |
| 444855007 | Arteriosclerosis of coronary artery bypass graft of transplanted heart (disorder) |
| 444856008 | Arteriosclerosis of internal mammary artery coronary artery bypass graft (disorder) |
| 446712002 | Thromboembolus of precerebral artery (disorder) |
| 713033007 | Dissection of artery of upper extremity (disorder) |
| 713081000 | Dissection of cerebral artery (disorder) |
| 716745004 | Livedo reticularis and cerebrovascular accident syndrome (disorder) |
| 719678003 | Non-obstructive atherosclerosis of coronary artery (disorder) |
| 721328009 | Injury of intracranial vessel of head (disorder) |
| 723869004 | Acute occlusion of aortoiliac artery co-occurrent and due to thromboembolus (disorder) |
| 723870003 | Acute occlusion of artery of lower limb co-occurrent and due to thromboembolus (disorder) |
| 723873001 | Arterial obstruction due to thrombotic embolism from mural thrombus of heart (disorder) |
| 723874007 | Arterial obstruction due to nonthrombotic embolism from heart (disorder) |
| 723875008 | Arterial obstruction due to thrombotic embolism from aneurysm of artery (disorder) |
| 724002003 | Retinal ischemia, digestive tract small vessel hyalinosis, diffuse cerebral calcification syndrome (disorder) |
| 724439005 | Microvascular embolism of arteriole (disorder) |
| 724993002 | Cerebral ischemic stroke due to occlusion of extracranial large artery (disorder) |
| 732230001 | Dissection of coronary artery (disorder) |
| 733126003 | Acute occlusion of artery of upper limb caused by thromboembolus (disorder) |
| 733199002 | Multifocal cerebral infarction due to and following procedure on cardiovascular system (disorder) |
| 21000119103 | Asymptomatic carotid artery stenosis (disorder) |
| 791000119109 | Angina associated with type II diabetes mellitus (disorder) |
| 1641000119107 | Coronary arteriosclerosis in native artery (disorder) |
| 5431000124100 | Thrombosis of anterior cerebral artery (disorder) |
| 5451000124107 | Thrombosis of superior cerebellar artery (disorder) |
| 5461000124109 | Thrombosis of anterior inferior cerebellar artery (disorder) |
| 5581000124100 | Thrombosis of posterior cerebral artery (disorder) |
| 5601000124105 | Thrombosis of posterior inferior cerebellar artery (disorder) |
| 9901000119100 | Occlusion of cerebral artery with stroke (disorder) |
| 10971000087107 | Myocardial ischemia during surgery (disorder) |
| 21631000119105 | Limb ischemia (disorder) |
| 99451000119105 | Cerebral infarction due to stenosis of carotid artery (disorder) |
| 116291000119103 | Occlusion of renal artery due to embolism (disorder) |
| 117051000119103 | Chronic total occlusion of coronary artery (disorder) |
| 139011000119104 | Coronary arteriosclerosis following coronary artery bypass graft (disorder) |
| 145891000119104 | Atherosclerosis of native arteries of the extremities (disorder) |
| 149821000119103 | Cerebral infarction due to carotid artery occlusion (disorder) |
| 284861000119104 | Atherosclerosis of bilateral carotid arteries (disorder) |
| 284871000119105 | Atherosclerosis of left carotid artery (disorder) |
| 284881000119108 | Atherosclerosis of right carotid artery (disorder) |
| 285141000119106 | Arteriosclerosis of autologous arterial coronary artery bypass graft (disorder) |
| 285151000119108 | Arteriosclerosis of autologous coronary artery bypass graft (disorder) |
| 285161000119105 | Occlusion of left carotid artery (disorder) |
| 285171000119104 | Occlusion of right carotid artery (disorder) |
| 285191000119103 | Left carotid artery stenosis (disorder) |
| 285201000119100 | Right carotid artery stenosis (disorder) |
| 291401000119102 | Spontaneous hemorrhage of subarachnoid space from left middle cerebral artery (disorder) |
| 429221000124104 | Dissecting aneurysm of cerebral artery (disorder) |
| 429811000124106 | Anterior cerebral artery embolism (disorder) |
| 429821000124103 | Posterior cerebral artery embolism (disorder) |
| 429831000124100 | Cerebellar artery embolism (disorder) |
| 429841000124105 | Superior cerebellar artery embolism (disorder) |
| 429851000124107 | Anterior inferior cerebellar artery embolism (disorder) |
| 429861000124109 | Posterior inferior cerebellar artery embolism (disorder) |
| 430721000124101 | Carotid artery occlusion without infarction (disorder) |
| 430731000124103 | Basilar artery occlusion without infarction (disorder) |
| 430831000124106 | Occlusion and stenosis of posterior cerebral artery without infarction (disorder) |
| 430851000124104 | Multiple AND bilateral precerebral artery stenosis with infarction (disorder) |
| 430861000124102 | Multiple AND bilateral precerebral artery stenosis without infarction (disorder) |
| 431391000124106 | Arteriosclerosis of aorta (disorder) |
| 431401000124108 | Arteriosclerosis of abdominal aorta (disorder) |
| 431411000124106 | Arteriosclerosis of thoracic aorta (disorder) |
| 431421000124103 | Arteriosclerosis of carotid artery (disorder) |
| 431431000124100 | Arteriosclerosis of renal artery (disorder) |
| 433821000124102 | Occlusion of internal carotid artery (disorder) |
| 433891000124100 | Cerebral infarction due to cerebral artery occlusion (disorder) |
| 433911000124103 | Cerebral infarction due to posterior cerebral artery occlusion (disorder) |
| 433931000124109 | Cerebral infarction due to internal carotid artery occlusion (disorder) |
| 433961000124100 | Cerebral infarction due to cerebral venous thrombosis (disorder) |
| 434151000124101 | Cerebral infarction due to anterior cerebral artery occlusion (disorder) |
| 434821000124104 | Vertebral artery occlusion with infarction (disorder) |
| 434831000124101 | Vertebral artery stenosis with infarction (disorder) |
| 434881000124100 | Multiple and bilateral precerebral arterial occlusion with infarction (disorder) |
| 434891000124102 | Internal carotid artery stenosis with infarction (disorder) |
| 434951000124104 | Middle cerebral artery occlusion (disorder) |
| 434961000124102 | Cerebral infarction due to middle cerebral artery occlusion (disorder) |
| 434991000124105 | Cerebral infarction due to basilar artery stenosis (disorder) |
| 435271000124103 | Vertebral artery occlusion without infarction (disorder) |
| 435281000124100 | Vertebral artery stenosis without infarction (disorder) |
| 436021000124100 | Carotid artery stenosis without infarction (disorder) |
| 436031000124102 | Anterior inferior cerebellar artery occlusion with infarction (disorder) |
| 436041000124107 | Posterior inferior cerebellar artery occlusion with infarction (disorder) |
| 436781000124104 | Intracranial artery occlusion with infarction (disorder) |
| 437911000124106 | Recurrent artery of Huebner occlusion with infarction (disorder) |
| 450251000124102 | Disorder of artery of upper extremity (disorder) |
| 451041000124103 | Atherosclerosis of coronary artery without angina pectoris (disorder) |
| 451361000124102 | Lipid-rich atherosclerosis of coronary artery (disorder) |
| 454031000124106 | Occlusion of superior cerebellar artery (disorder) |
| 691471000119109 | Ischemia co-occurrent and due to increased oxygen demand (disorder) |
| 11018701000119100 | Coronary arteriosclerosis after percutaneous coronary angioplasty (disorder) |
| 15712481000119100 | Ischemia of left lower extremity (disorder) |
| 15712521000119100 | Ischemia of right lower extremity (disorder) |
| 15712561000119100 | Ischemia of bilateral lower limbs (disorder) |
| 15712601000119100 | Ischemia of bilateral upper limbs (disorder) |
| 15712681000119100 | Ischemia of left upper extremity (disorder) |
| 15960061000119100 | Unstable angina co-occurrent and due to coronary arteriosclerosis (disorder) |
| 15960141000119100 | Angina co-occurrent and due to coronary arteriosclerosis (disorder) |
| 15960781000119100 | Angina co-occurrent and due to arteriosclerosis of autologous arterial coronary artery bypass graft (disorder) |
| 15966701000119100 | Atheroembolism of right renal artery (disorder) |
| 15966741000119100 | Atheroembolism of bilateral renal arteries (disorder) |
| 15966781000119100 | Atheroembolism of left renal artery (disorder) |
| 16002751000119100 | Pain of bilateral upper limbs co-occurrent and due to ischemia (disorder) |
| 16009351000119100 | Atheroembolism of left upper limb (disorder) |
| 16009391000119100 | Atheroembolism of right upper limb (disorder) |
| 16012471000119100 | Atheroembolism of left lower limb (disorder) |
| 16012631000119100 | Atheroembolism of right lower limb (disorder) |
